# Promoting Well-Being in Old Age: The Psychological Benefits of Two Training Programs of Adapted Physical Activity

**DOI:** 10.3389/fpsyg.2018.00828

**Published:** 2018-05-28

**Authors:** Antonella Delle Fave, Marta Bassi, Elena S. Boccaletti, Carlotta Roncaglione, Giuseppina Bernardelli, Daniela Mari

**Affiliations:** ^1^Department of Pathophysiology and Transplantation, Università degli Studi di Milano, Milan, Italy; ^2^Department of Biomedical and Clinical Sciences “L. Sacco”, Università degli Studi di Milano, Milan, Italy; ^3^Fondazione IRCCS Ca' Granda Ospedale Maggiore Policlinico (IRCCS), Milan, Italy; ^4^Department of Clinical Sciences and Community Health, Università degli Studi di Milano, Milan, Italy

**Keywords:** aging, adapted physical activity, intervention programs, well-being, emotion regulation strategies

## Abstract

In the last few decades, the relationship between physical conditions and mental health has increasingly attracted the interest of researchers and professionals across disciplines. This relationship is especially relevant in old age, as the challenges posed by aging at various levels represent crucial concerns for policy makers. Due to the remarkable increase in life expectancy across countries, sustainable prevention strategies are needed to help individuals preserve psychophysical well-being in old age. In particular, the regular practice of a moderately intense physical activity is recommended by the World Health Organization to enhance balance, prevent falls, strengthen muscles, and promote psychophysical well-being. Daily physical exercise represents a beneficial and low-cost strategy, easily accessible to the general population and potentially customizable to specific needs through brief training programs. Based on these premises, the present research aimed at longitudinally evaluating mental well-being among 58 Italian people aged 67–85, who were involved in two Adapted Physical Activity (APA) training programs. Inclusion criteria for participation comprised high autonomy levels in daily activities, no cognitive impairment, sedentary habits or only occasional performance of moderate physical activity. Based on physical and functional assessment, 39 participants joined a program of adapted motor activity (PoliFit; Study 1), while 19 participants attended a variant program specifically designed for people with osteoporosis (OsteoFit; Study 2). Well-being dimensions were assessed through the Mental Health Continuum-Short Form, the Emotion Regulation Questionnaire and the Satisfaction with Life Scale. Physical functioning were evaluated before and after the programs through the Short Physical Performance Battery and the Handgrip Dynamometer Jamar Test. Findings highlighted that, besides physical benefits, participants reported significantly more adaptive emotion regulation strategies after both training programs; in addition, participants attending OsteoFit reported significantly higher levels of emotional well-being. Results suggest the potential of moderate physical activity in promoting mental health, emphasizing the additional role of training programs as cost-effective opportunities for elderly people to socialize and improve emotional functioning. Overall, the findings support the view of old age as a stage of competence development and adaptive adjustment, rather than a phase of mere psychophysical decline.

## Introduction

The Ottawa Charter for Health Promotion (WHO, 1986) provides guidelines for a global social action aimed at promoting citizens' psychophysical well-being. According to the Charter, health promotion initiatives should foster individuals' active control over their own health conditions, and personal engagement in improving them.

The demographic and epidemiological changes that took place in the last few decades—including longer life expectancy, reduction in birth rates and childhood mortality, as well as increase in the number of years spent with chronic diseases (often related to unhealthy lifestyles)—have made the Ottawa guidelines a necessary reference point for policy makers and health professionals (Kickbusch, 2003). In particular, the medical, social, and financial challenges posed by such a massive epidemiological transition call for a substantial revision of the healthcare systems' strategies and underlying conceptual model. The biomedical and disease-focused approach needs to be replaced by a more comprehensive perspective, focusing on bio-psycho-social well-being, and prevention at all levels (primary, secondary, and tertiary). A core resource to achieve these goals is the promotion of citizens' empowerment, active involvement, responsibility and self-determination in preserving and improving their own health conditions (Ng et al., 2012; Jeste and Palmer, 2013).

Well-being as the focus of healthcare interventions is a complex construct, encompassing different psychological and social dimensions (Prilleltensky, 2005). Researchers' efforts to identify and quantify subjective indicators of well-being led to the development of different theoretical models and measurement tools, whose adoption proved to be effective in a variety of research and intervention programs. Well-being is defined as hedonic, or subjective, when it is identified and measured as the global perception of life satisfaction, combined with the predominance of positive over negative affect in daily life (Kahneman et al., 1999). From the theoretical perspective of eudaimonia, instead, well-being is conceptualized and measured as a dynamic process including multiple indicators, that have been variably combined in different models (Huta and Waterman, 2014). The eudaimonic model most widely used in clinical interventions is Psychological Well-Being (Ryff, 1989), consisting of six dimensions: self-acceptance, mastery of the environment, autonomy, positive relationships, personal growth, and life purposes. A further broadening of the eudaimonic perspective was represented by the formalization of Social Well-Being (Keyes, 1998), a construct including the dimensions of perceived social coherence, actualization, integration, acceptance, and contribution.

During the last decade, researchers have attempted to build more comprehensive models, integrating hedonic and eudaimonic dimensions in a meaningful way. A promising outcome of these attempts is the Mental Health Continuum (MHC; Keyes, 2007), a model of optimal human functioning encompassing emotional well-being (including positive affect and life satisfaction), the six dimensions of psychological well-being, and the five facets of social well-being. The condition of individuals reporting high ratings in the majority of these dimensions is defined as flourishing, and it corresponds to high mental health. Conversely, low scores in emotional, psychological and social well-being correspond to the condition of languishing, a state of disengagement, negative affect and social withdrawal whose negative physical and psychosocial consequences have been highlighted in a variety of studies (Keyes, 2005). Although languishing differs from mental illness, whose presence is based on a specific set of symptoms, it is associated with higher risk for developing affective disorders (Keyes, 2007).

### Successful aging: subjective dimensions

The epidemiological transition described above has brought to the attention of researchers, professionals and policy makers the importance to promote physical health and psychosocial well-being among elderly citizens, whose percentage is progressively increasing worldwide, especially in Europe. Resource investments are necessary to adequately address the needs of people in this life stage, but also to foster their empowerment and active engagement in health promoting behaviors (Prochaska and Prochaska, 2016).

Studies on well-being promotion in old age revolve around the concept of successful aging (Depp and Jeste, 2006; Doyle et al., 2010). This concept was previously based on objective indicators such as absence of physical impairments, cognitive disabilities, and social restrictions (Rowe and Kahn, 1997); more recently it was expanded to include positive psychological indicators such as wisdom, a complex construct encompassing emotional stability, rational decision-making based on life knowledge, empathy and compassion (Jeste and Harris, 2010; Ardelt, 2016). Notwithstanding the substantial physical and social changes occurring with aging, the subjective attitude toward these changes plays a central role in predicting well-being and physical health in old age (Levy and Myers, 2004; Tovel and Carmel, 2014; Bryant et al., 2016; Thomsen et al., 2017).

Research studies explored the specific role of hedonic and eudaimonic well-being components in promoting successful aging. High levels of subjective well-being foster physical health and longevity (Chida and Steptoe, 2008; Koopmans et al., 2010; Diener and Chan, 2011); high levels of psychological well-being can counterbalance the negative consequences of chronic disease and disabilities (Bassi et al., 2014; Ryff, 2014). Flourishing people report the lowest incidence of chronic disease, and the highest probability of 10-year survival (Keyes et al., 2010). The English Longitudinal Study of Ageing (ELSA) showed that subjective well-being is associated with higher survival rates (Steptoe and Wardle, 2011), even though this pattern is exposed to variations across nations (Steptoe et al., 2015). A seminal study in this domain showed that overall, despite worse physical conditions, older people are happier and more satisfied with their life than younger ones (Blanchflower and Oswald, 2008). As concerns psychological well-being, some studies conducted in the US detected aging related changes, including a decrease in personal growth and purpose in life, an increase in autonomy and environmental mastery, and stable values of positive relations and self-acceptance (Clarke et al., 2000; Keyes et al., 2002). In a recent longitudinal study, however, these findings were confirmed only for mastery, while more complex trends emerged for the other dimensions (Springer et al., 2011). As for social well-being, among US participants aging was associated with higher values of social acceptance and integration, while contribution and coherence were higher among younger participants (Keyes and Shapiro, 2004).

Findings from a recent cross-sectional study, involving Canadian participants in three different life stages, further confirmed the association of aging with higher levels of emotional well-being and autonomy, and with lower levels of personal growth and purpose (Mackenzie et al., 2017). The age related increase in perceived autonomy, mastery, and emotional well-being is consistent with longitudinal findings from Sweden, showing that perceived internal locus of control and emotional stability moderate the impact of serious chronic diseases on satisfaction with life among elderly people (Berg et al., 2011). Analogously, high levels of perceived control and the preferential use of adaptive coping strategies (problem focused and positive reappraisal coping) are associated with wisdom and both hedonic and eudaimonic well-being (Etezadi and Pushkar, 2013).

The recurrent evidence of higher emotional stability in old age is confirmed by studies showing that, compared with younger participants, older adults report fewer negative emotions (Mroczek, 2001) and show preference for low arousal positive emotions (Mogilner et al., 2011). These emotional patterns have been interpreted through the lens of socioemotional selectivity theory (Carstensen and Mikels, 2005), according to which aging leads individuals to place increasing value on emotionally meaningful goals rather than on long-term, cognitively demanding ones. This preferential resource investment promotes more effective emotion regulation, defined as the process through which individuals control their emotional experiences and expressions (Gross et al., 1997; Carstensen et al., 1998; Gross, 1998).

Emotion regulation comprises two strategies: cognitive reappraisal and suppression. Cognitive reappraisal allows individuals to modify their evaluation of a potentially emotion-laden situation by reducing negative emotions or increasing positive ones. It represents an antecedent-focused strategy, as it is enacted before the onset of the psycho-physical and behavioral changes related to the emotional response. Suppression is instead a response-focused strategy, as it inhibits emotional expression after the emotion onset (Gross and Levenson, 1993). While reappraisal is adaptive, as it enhances control and buffering of emotional responses, suppression of emotional behaviors may lead to an increase in emotional intensity at the experiential level, with negative consequences in terms of perceived control, as well as cognitive and executive functioning (Gross, 2002; Gross and John, 2003; John and Gross, 2004; Franchow and Suchy, 2017). Moreover, during social interactions suppression inhibits expressive signals relevant to information exchange, and it leads individuals to focus on the monitoring of their own emotions, rather than on the information coming from their interlocutor (Butler et al., 2003). Studies comparing participants at different life stages highlighted that older adults report more frequent use of cognitive reappraisal and lower use of suppression than adolescents and young adults (Urry and Gross, 2010).

Gender differences in emotion regulation strategies were also identified. Across life stages, women endorse a wider variety of cognitive coping strategies compared to men (Martin and Dahlen, 2005), and they more often use reappraisal based ones, while men seem to find greater difficulties in developing a positive or accepting attitude toward aging related problems such as illness or loss (Nolen-Hoeksema and Aldao, 2011). Among women, acceptance of outcomes and attribution of a positive meaning to life events is associated with lower levels of worry (Zlomke and Hahn, 2010).

### Successful aging: objective and contextual aspects

Several studies conducted in different countries have investigated the role of objective dimensions as predictors of successful aging. The most relevant ones are represented by physical health (Angner et al., 2013), social support from family and friends (Dai et al., 2013; Dumitrache et al., 2015), free time investment (Adams et al., 2011; Brajsa-Zganec et al., 2011; Lee et al., 2014), and physical activity (Klusmann et al., 2012; Ku et al., 2016).

For the purpose of this study, free time and physical activity deserve a more focused examination. Leisure activities, especially the so called structured or serious ones such as arts and crafts, volunteering, sports, hobbies, attending cultural events, and reading (Stebbins, 2007), are important predictors of both hedonic and eudaimonic well-being (Delle Fave and Bassi, 2003; Lee et al., 2014; Kuykendall et al., 2015). These activities provide individuals with opportunities for engaging in autonomously chosen tasks, consistent with the person's interests and self-determined goals, and for experiencing positive emotions, competence enhancement and mastery, as well as social integration and connectedness (Sotgiu et al., 2011; Bassi and Delle Fave, 2013; Toepoel, 2013; Kekäläinen et al., 2017).

As concerns physical activity, besides providing health benefits it promotes the psychosocial well-being of citizens in the later life stage (Lampinen et al., 2006; Moran et al., 2014; Awick et al., 2015). Studies investigating the effects of physical activity programs addressed to older people highlighted their positive impact on adjustment to aging through the promotion of physical health (Battaglia et al., 2016), self-determination (Klusmann et al., 2012; Mack et al., 2017), and social connectedness (Ku et al., 2016).

### The present research

In the psychological literature, well-being among elderly people is assessed through a variety of instruments. Research in this domain, however, predominantly includes cross-sectional studies on healthy participants, and longitudinal surveys on large samples based on simplified well-being measures, often consisting of single items. A laudable exception is represented by the Midlife in the United States Study (MIDUS), subsequently launched also in Japan (MIDJA), whose set of questions includes several hedonic and eudaimonic well-being measures. Studies thoroughly investigating the psychological outcomes of physical activity and exercise interventions addressed to elderly participants are less frequent, especially in the biomedical domain, where quality of life measures are often used as measures of psychological functioning (Windle et al., 2010; Giangregorio et al., 2015). The present research is therefore aimed at contributing to the scanty literature investigating specific dimensions of well-being in relation to interventions based on physical activity addressed to the elderly population.

The implementation of interventions aimed at promoting health and well-being among older citizens is particularly needed in Italy, currently representing the oldest country in the world according to the aging index (measured as the ratio between the percentages of citizens above 65 and below 15). Italians above 65 account for 22.3% of the population, and the overall average age is 44.9 years (ISTAT, 2017). Intervention programs addressed to elderly citizens are therefore a major goal in the domain of public health; moreover, there is growing awareness of the need for evaluating their outcomes not only in terms of physical health, but also at the level of psychological functioning.

Based on these premises, the two studies included in this work aim at evaluating the psychological outcomes of two different training programs of Adapted Physical Activity (APA) for people aged over 65 with sedentary habits. APA is an umbrella term referring to physical activities and sports that are designed taking into account the interests and capabilities of persons with limiting conditions, including disabilities, health impairments, or aging (Doll-Tepper et al., 1990; Hutzler and Sherrill, 2007; Battaglia et al., 2016). The overall goal of the APA programs implemented in the two studies was the prevention of physical and functional decay and the promotion of healthy aging, through sessions of aerobic, strength, balance, coordination, and flexibility exercises. However, the two studies differed as concerns the typology of participants. Participants in Study 1 were in overall good psychophysical health conditions, and were independent in Activities of Daily Living (ADL). Participants in Study 2 were free from cognitive impairments and were independent in ADL, but they had been diagnosed with osteoporosis; therefore, the training program was specifically tailored to address the physical challenges related to this pathology, characterized by reduced bone density and higher risk of fractures. Osteoporosis affects over 200 million people worldwide, predominantly women in the post-menopausal stage, and it is becoming a major public health concern, due to the related risk of mobility impairments, comorbidities, and permanent disabilities (Hernlund et al., 2013). The specific aim of the APA program designed for participants in Study 2 was to improve balance and proprioception, as well as promote muscle strengthening in order to prevent falls.

As the major aim of the current research was to investigate well-being, both studies were focused on the assessment of psychological variables. In light of the importance of emotion regulation in aging (Carstensen and Mikels, 2005), attention was paid to the assessment of this dimension. Measures of the emotional, psychological and social components of well-being, as well as of satisfaction with life were also included. Based on previous findings, highlighting the positive impact of physical activity on the well-being of aging individuals (Moran et al., 2014; Awick et al., 2015), we expected to identify improvements in participants' levels of well-being, especially at the emotional and social levels.

## Study 1

### Methods

#### Participants and recruitment

Participants in Study 1 were 39 elderly persons (34 women and 5 men) aged between 68 and 85 years (*M* = 74.05, *SD* = 4.56) who were recruited at C.A.M. centers^1^ in Milano, Northern Italy. Inclusion criteria were voluntary participation in the study; low levels of physical activity (i.e., less than 20 min of regular physical exercise once a week over the last few weeks); and ability to walk 400 m in max. 15 min with no need to sit down or lean and require assistance of another person or a walker (i.e., walk test). Exclusion criteria were dependence in Activities of Daily Living according to Katz, Barthel, and Lawton Indexes (Katz et al., 1963; Mahoney and Barthel, 1965; Lawton and Brody, 1969); cognitive impairment with a score <24 at the Mini Mental State Examination (MMSE; Folstein et al., 1975); being under anticholinesterase treatment; a diagnosis of Parkinson's disease or being under anti-Parkinson medicament; current participation in rehabilitation programs; psychiatric disorders; cardiorespiratory diseases with contraindications for moderate physical activities; quoad vitam prognosis <1 year.

On average, participants' ADL independence ratings amounted to 5.67 (*SD* = 0.48) at the Katz Index, 99.15 (*SD* = 1.35) at the Barthel Index, and 8 (*SD* = 0) at the Lawton Index, globally highlighting a high degree of autonomy and ADL functioning. Participants also reported good cognitive functioning, with an MMSE score of 28.72 (*SD* = 1.53). Concerning education, 59% of participants had a high school degree, 21% had attended elementary and middle school, and 21% had a college degree. Regarding demographic characteristics, 56.4% of participants lived with their partner (81.8%) or other family members such as children (18.2%), while 43.6% lived alone. The majority (74.4%) had children (*M* = 1.69, *SD* = 0.66). Table 1 includes additional self-reported information about leisure time activities and participation in associations.

**Table 1:** Percentage distribution of leisure activities and association membership among participants engaged in Polifit and Osteofit programs.

	**Study 1 PoliFIT** **program** **%**	**Study 2 OsteoFIT** **program** **%**
**Leisure Activities**	**(*****N***_p_ = **32;** ***N***_a_ = **75)**	**(*****N***_p_ = **13;** ***N***_a_ = **22)**
Sports	17.3	9.1
Arts and crafts	20.0	18.1
Volunteering	13.3	4.5
Family/social interactions	4.0	–
Attending cultural events	12.0	36.4
Intellectual activities	13.3	9.1
Media	2.7	4.5
Shopping	1.3	–
Yoga, meditation	4.0	9.1
Outdoor activities	12.0	9.0
**Associations**	**(*****N***_p_ = **20;** ***N***_a_ = **26)**	**(*****N***_p_ = **7;** ***N***_a_ = **8)**
Sports	3.8	–
Culture	46.1	62.5
Social/healthcare volunteering	26.9	37.5
Religious organizations	11.5	–
Politics	11.5	–

*N_p_, number of participants; N_a_, number of answers; –, no answer reported*.

The majority of participants (82.1%) reported having one or more leisure time activity, primarily intellectual and sedentary ones, such as reading, volunteering, arts and crafts, and attendance in cultural events. Sports accounted for less than 20% of the answers. A lower percentage of participants (51.3%) also reported taking active part in associations, primarily cultural ones.

#### Protocol and procedure

The research protocol was approved by the Ethical Committee of Milano area B (document no. 813/2015). A group of 187 older adults aged 67–89 volunteered for the study. After presenting the research and obtaining written informed consent, participants' current cognitive and physical functioning was assessed by a geriatrist at the hospital, in order to appraise eligibility. After assessment, 13 volunteers did not meet the study criteria for the following reasons: impairment in the walk test (*N* = 7 participants); functional disabilities (*N* = 2); MMSE score < 24 (*N* = 1); psychiatric disorders (*N* = 1); intense physical activity (*N* = 1); heart disease (*N* = 1). In addition, two persons could not be enrolled in the training because of severe hearing impairment, and one because of severe osteoporosis. Due to organizational and space constraints, the program was segmented in multiple waves over time. The first wave, to which the current study refers, included 60 participants: 39 completed the study, 10 dropped out, and 11 are currently on a waiting list for future waves of the training program. Participants who dropped out were comparable in terms of demographic features to those who completed the study. They were 8 women and 2 men, aged between 71 and 78 years (*M* = 74.10, *SD* = 3.11), with a mean score of 28.40 (*SD* = 1.43) at the MMSE, 5.70 (*SD* = 0.48) at the Katz Index, 99.60 (*SD* = 0.84) at the Barthel Index, and 8 (*SD* = 0) at the Lawton Index.

Before the start of the training program (T0), participants provided personal information on their socio-demographic characteristics and leisure time activities. They further filled in a battery of psychological questionnaires and performed physical tests measuring the variables of interest in this study (see Materials section below).

Participants then joined the first wave of the *PoliFIT Program*, consisting of group sessions of structured and adapted physical activities (APA), taking place in a dedicated gym at the Research and Clinical Institute Fondazione Ca' Granda Ospedale Policlinico in Milano. The activities had been planned by a team of specialized physicians and physiotherapists, with the aim to prevent physical and functional decay, and to promote healthy aging. Participants were randomly divided into groups of 10 individuals each, attending weekly sessions of 45 min for 4 months. The sessions were run by two physiotherapists. The program consisted of moderate or intense physical activity including aerobic, strength, balance, coordination, and flexibility exercises, with the use of sticks, elastic bands, balance platforms, soft carpets, and rehabilitative balls. On average, participants' rate of attendance was 77.35% (*SD* = 13.27).

At the end of the program (T1), participants were again invited to fill in the same battery of psychological questionnaires and perform the physical tests administered at T0.

#### Materials

Information was collected from participants about age, gender, education, family status (“whom do you live with?”, “do you have children? If yes, how many?”)^2^, as well as involvement in leisure time activities and in associations.

##### Screening assessment

The *Mini Mental State Examination* (Folstein et al., 1975) is an extensively-used 30-item questionnaire measuring presence and severity of cognitive impairment. Scores range from 0 to 30, with ratings ≥ 24 indicating normal cognition, 19–23 mild, 10–18 moderate, and ≤ 9 severe cognitive impairment.

The *Katz Index* (Katz et al., 1963) and the *Barthel Index* (Mahoney and Barthel, 1965) are two instruments assessing individuals' abilities to independently perform activities of daily living. Scores range from 0 to 6 for Katz index (with 6 indicating full function, 4 moderate impairment, 2 or less severe functional impairment), and from 0 to 100 for Barthel Index (0–20 indicating total dependence, 21–60 severe dependence, 61–90 moderate, and 91–100 full independence).

The *Lawton Index* (Lawton and Brody, 1969) measures participants' independence in eight instrumental activities: using the telephone, shopping, preparing food, housekeeping, doing the laundry, using transportation, handling medications and finances. Scores span from 0 “full dependence” to 8 “full independence.”

##### Psychological assessment

The *Mental Health Continuum Short Form* (MHC-SF; Keyes, 2005; Italian version Petrillo et al., 2015) measures participants' mental health reported over the last month on 6-point scales: 0 (never), 1 (once or twice a month), 2 (about once a week), 3 (about 2 or 3 times a week), 4 (almost every day), 5 (everyday). It comprises 14 items, of which 3 measure the frequency of emotional well-being (EWB), 6 psychological well-being (PWB), and 5 social well-being (SWB). Sample items are “During the past month, how often did you feel”: “happy” (EWB), “that you had experiences that challenged you to grow and become a better person” (PWB), “that you had something important to contribute to society” (SWB). The items for each dimension were summed to their total score, with higher ratings indicating greater levels of well-being. Cronbach's alphas at T0 were 0.50 for EWB, 0.62 for PWB, and 0.70 for SWB; at T1 they were 0.67 for EWB, 0.76 for PWB, and 0.71 for SWB.

The *Emotion Regulation Questionnaire* (ERQ, Gross and John, 2003; Italian version Balzarotti et al., 2010) is a 10-item scale designed to measure respondents' tendency to regulate their emotions through Cognitive Reappraisal (CR; 6 items) and Expressive Suppression (ES; 4 items) on 7-point Likert-type scales ranging from 1 (strongly disagree) to 7 (strongly agree). Sample items are “When I want to feel more positive emotion (such as joy or amusement), I change what I'm thinking about,” and “When I am feeling positive emotions, I am careful not to express them,” respectively. The items for each emotion regulation strategy were summed to their total score, with higher ratings indicating greater strategy use. At T0 Cronbach's alphas amounted to 0.67 for both CR and ES; at T1 they were 0.69 for CR and 0.75 for ES.

The *Satisfaction With Life Scale* (SWLS, Diener et al., 1985; Italian version Di Fabio and Palazzeschi, 2012) asks respondents to rate their level of overall life satisfaction from 1 (strongly disagree) to 7 (strongly agree) on 5 statements such as “The conditions of my life are excellent.” A total summed score was calculated, with higher scores accounting for higher life satisfaction. Cronbach's alphas were 0.88 at T0 and 0.92 at T1.

Physical assessments at T0 and T1 were performed through the *Short Physical Performance Battery* (SPPB, Guralnik et al., 1994) and the *Handgrip Strength Test* (Han et al., 2011), as reported by Bernardelli et al. (2017).

#### Data analysis

Analyses were performed using software SPSS 24. After data screening, descriptive statistics were used to report participants' sociodemographic characteristics and physical functioning, and to illustrate study variables before and after the PoliFIT intervention (respectively, T0 and T1). In addition, a categorical diagnosis of mental health at T0 and T1 was calculated for each participant based on their scores at the MHC-SF (Keyes, 2005). A diagnosis of flourishing was made if someone felt 1 of the 3 emotional well-being symptoms and 6 of the 11 psychological and social well-being symptoms “every day” or “almost every day” in the past month. A diagnosis of languishing was made if someone felt 1 of the 3 emotional well-being symptoms and 6 of the 11 psychological and social well-being symptoms “never” or “once or twice” in the past month. Participants who were neither languishing nor flourishing were diagnosed with moderate mental health. One-way repeated ANOVA was applied to compare scores in the psychological and physical variables before and after the program. The strength of effect sizes was evaluated through partial eta squared ($\eta_{\text{p}}^{2}$) with 0.01 indicating a small, 0.06 a moderate, and 0.14 a large effect (Cohen, 1988).

### Results

Participants' mean ratings in the psychological measures at T0 (prior to intervention) and T1 (following the intervention) are reported in Table 2.

**Table 2:** Mean values of the psychological dimensions before (T0) and after (T1) the PoliFIT and OsteoFIT Programs.

	**Study 1** **PoliFIT program**				**Study 2** **OsteoFIT program**			
	***N***	***M***	***SD***	**Range**	***N***	***M***	***SD***	**Range**
**MENTAL HEALTH CONTINUUM-SF**								
**Emotional well-being**								
T0	39	9.64	2.50	4–14	19	9.68	2.73	5–13
T1	39	9.87	2.88	2–15	19	11.05	1.87	8–15
**Social well-being**								
T0	39	9.67	4.43	3–19	19	10.16	5.53	2–21
T1	39	10.62	4.63	2–21	19	12.58	5.86	3–21
**Psychological well-being**								
T0	39	22.23	4.20	13–29	19	22.16	4.51	12–29
T1	39	22.10	4.40	13–30	19	23.84	3.90	12–29
**Satisfaction with life scale**								
T0	39	23.21	6.19	9–33	19	22.89	7.01	8–34
T1	39	23.43	7.01	5–34	19	24.46	5.76	11–35
**EMOTION REGULATION QUESTIONNAIRE**								
**Cognitive reappraisal**								
T0	39	28.89	6.16	15–40	19	30.78	6.80	20–42
T1	39	31.79	5.00	20–42	19	32.02	5.43	21–42
**Expressive suppression**								
T0	39	14.97	5.25	5–25	19	18.16	4.88	8–28
T1	39	15.21	5.90	4–28	19	15.26	7.12	4–25

Considering the scale ranges, participants reported at both times high well-being scores, especially in emotional and psychological dimensions, while social well-being ratings were somewhat low. In addition, the categorical diagnosis of mental health revealed that at T0 71.8% of participants (*N* = 28) had moderate mental health and 28.2% were flourishing (*N* = 11). At T1, 66.7% reported moderate mental health (*N* = 26), 30.8% were flourishing (*N* = 12), and one participant was languishing. Concerning satisfaction with life, based on normative criteria (Pavot and Diener, 2009), participants' scores were in line with those from a group of older American adults (*M* = 24.2 *SD* = 6.9) and fell within the range 21–25, indicating slight satisfaction. Finally, in terms of emotion regulation strategies, participants tended to use more cognitive reappraisal and less expressive suppression.

At T1, all participants showed significant improvements in their physical performance, for both upper and lower limbs (Bernardelli et al., 2017). One-way repeated ANOVAs were performed to compare scores at T0 and T1 for psychological variables. Analyses revealed a significant large time effect for cognitive reappraisal [Wilks' λ = 0.77, *F*_(1, 38)_ = 11.02, *p* = 0.002, $\eta_{p}^{2}$ = 0.22], showing an increase in the use of this emotion regulation strategy from before to after the training program.

### Brief discussion

Study 1 was conducted in order to evaluate the psychological outcomes of an APA training program in a group of older adults in overall good health conditions, but showing sedentary habits both in daily life and in the typology of leisure activities usually practiced. Through weekly group sessions of structured activities, the program was aimed at promoting a healthier and more active lifestyle, whose positive role as a protective factor against physical decay and related comorbidities has been repeatedly demonstrated (Lampinen et al., 2006; Battaglia et al., 2016). On average, at T0 participants reported a good level of mental health: None was languishing, and over one fourth were flourishing; their most frequent emotion regulation strategy was the adaptive one of cognitive reappraisal; and their life satisfaction levels were comparable to those reported by US older adults. Besides producing physical benefits (Bernardelli et al., 2017), the training program led to a further improvement in participants' well-being, through an increase in the use of cognitive appraisal at T1, while the values of the other well-being indicators remained substantially stable.

This result is relevant, as several studies have revealed the positive relationship between cognitive appraisal and psychological benefits for older adults. In particular, this emotion regulation strategy represents a protective factor, allowing individuals to find meaning and positive aspects in the management of physical illness—an aging-related event to which older adults are physiologically more exposed (Zlomke and Hahn, 2010; Nowlan et al., 2015). It is also worth noticing that participants were perseverant in their engagement in the training program, as highlighted by the high attendance rate.

## Study 2

### Methods

#### Participants and recruitment

Participants in Study 2 were 19 elderly persons who were recruited at the bone metabolism surgery of the Research and Clinical Institute Fondazione Ca' Granda Ospedale Policlinico in Milano. Inclusion criteria were voluntary participation in the study; being positive at the Romberg Test for disequilibrium; bone density test showing a *T*-score < −2.5, or vertebral fragility fractures (after recovery and rehabilitation). Exclusion criteria were cognitive impairment with a score < 24 at the Mini Mental State Examination (MMSE); diagnosis of Parkinson's disease or being under anti-Parkinson medicament; otovestibular diseases; severe visual impairment; EMG-verified peripheral neuropathies; severe brain injuries; severe heart diseases (NYHA > 3); severe pulmonary disease (Chronic Obstructive Pulmonary Disease; COPD) requiring oxygen therapy; current participation in rehabilitation programs; psychiatric disorders; quoad vitam prognosis < 1 year.

Participants were 18 women and 1 man aged between 67 and 84 years (*M* = 76.58, *SD* = 5.31). On average, they reported good cognitive functioning, with an MMSE score of 27.42 (*SD* = 1.83). Concerning education, 64.7% had a high school degree, 29.4% had attended elementary and middle school, and 5.9% had a college degree. At the demographic level, 63.2% lived alone, while 36.8% lived with their partner (71.4%) or other family members such as children (28.6%). The majority (73.7%) had children (*M* = 1.64, *SD* = 0.63). As shown in Table 1, 68.4% of participants reported having one or more leisure activities, substantially sedentary; the most frequent one was attendance in cultural events. A lower percentage of participants (36.8%) also reported taking active part in associations, primarily cultural ones. Sports were reported in a very limited percentage, but it is important to consider that intense physical exercise is not recommended for persons with osteoporosis.

#### Protocol and procedure

The research protocol was approved by the Ethical Committee of Milano area B (document no. 786/2016). A group of 24 participants volunteered in the study. After the research presentation, they provided written informed consent. Then, participants' current physical functioning was assessed by a geriatrist and physiotherapists at the hospital, in order to control for inclusion/exclusion criteria. All participants were deemed eligible for the study: 19 completed the study and 5 dropped out. The latter group included 5 women, aged between 71 and 83 (*M* = 77.80, *SD* = 5.89), with a mean MMSE score of 27.80 (*SD* = 2.17). Like in Study 1, before the start of the training program (T0) participants provided personal information, filled in a battery of psychological questionnaires and performed physical tests measuring the variables of interest, as reported in the Materials section.

Participants then joined the *OsteoFIT Program*, consisting of (1) active structured and adapted physical activities (APA) and (2) exercises to be performed with equipment for aerobic and strength training. The protocol targeted different muscle groups and was designed by a team of specialized physicians and physiotherapists, with the aim to improve balance and proprioception, and to promote muscle strengthening. Participants were randomly divided into two groups of up to 10 individuals. For 14 consecutive weeks, they attended two 45-min training sessions a week at the hospital gym. One of the weekly sessions consisted of moderate intensity activity, including aerobic, balance, coordination, and flexibility exercises with the use of sticks, balance platforms, soft carpets and rehabilitative balls. The other session consisted of a strength training for both lower and upper limbs by means of Technogym® machines. Participants' average rate of attendance amounted to 81.68% (*SD* = 14.11). At the end of the program (T1), participants were again invited to complete the battery of psychological questionnaires and perform the physical tests administered at T0.

#### Materials

Information was collected on age, gender, education, family status^3^, as well as involvement in leisure time activities and in associations. Both at T0 and T1, participants filled out the same assessment tools applied and described in Study 1. More specifically, The *Mental Health Continuum Short Form* (MHC-SF; Keyes, 2005; Petrillo et al., 2015) was administered to appraise participants' mental health in its emotional, psychological and social components. Cronbach's alphas at T0 were 0.58 for EWB, 0.81 for PWB, and 0.74 for SWB; at T1 they were 0.71 for EWB, 0.67 for PWB, and 0.81 for SWB. The *Emotion Regulation Questionnaire* (ERQ, Gross and John, 2003; Balzarotti et al., 2010) measured respondents' use of Cognitive Reappraisal (CR) and Expressive Suppression (ES) as emotion regulation strategies. At T0 Cronbach's alphas amounted to 0.89 for CR and 0.65 for ES; at T1 they were 0.58 for CR and 0.74 for ES. The *Satisfaction With Life Scale* (SWLS, Diener et al., 1985; Di Fabio and Palazzeschi, 2012) assessed participants' level of overall life satisfaction. Cronbach's alphas were 0.92 at T0 and 0.84 at T1.

In addition to these psychological measures, participants' physical functioning was assessed through the Short Physical Performance Battery (SPPB, Guralnik et al., 1994) and the Handgrip Strength Test (Han et al., 2011) as in Study 1. These data are still at the coding stage.

#### Data analysis

Analyses in Study 2 were similar to the ones performed in Study 1. Using software SPSS 24, descriptive statistics were first calculated for participants' sociodemographic characteristics, and for the psychological outcome variables before and after the OsteoFIT intervention (respectively, T0 and T1). In addition, the categorical diagnosis of mental health at T0 and T1 was calculated for each participant based on their scores at the MHC-SF (Keyes, 2005). One-way repeated ANOVA was finally applied to compare scores of the psychological variables before and after the program. The strength of effect sizes was evaluated through partial eta squared ($\eta_{\text{p}}^{2}$) with 0.01 indicating a small, 0.06 a moderate, and 0.14 a large effect (Cohen, 1988).

### Results

Table 2 illustrates participants' mean ratings in the psychological measures at T0 (before the program) and T1 (after the program). As concerns mental health, assessed through MHC-SF, considering the scale ranges high scores were reported at both times for the emotional and psychological dimensions of well-being, while social well-being ratings were rather low. The categorical diagnosis of mental health revealed that at T0 73.7% of participants (*N* = 14) had moderate mental health and 26.3% were flourishing (*N* = 5), while at T1 52.6% reported moderate mental health (*N* = 10), and 47.4% were flourishing (*N* = 9). No participants were classified as languishing, both at T0 and T1. Overall, participants reported being slightly satisfied with life (SWLS range 21–25; Pavot and Diener, 2009); scores were in line with those from a normative group of older American adults (*M* = 24.2 *SD* = 6.9). Finally, as regards emotion regulation, assessed through ERQ, participants reported high use of cognitive reappraisal at both times and a lower use of expressive suppression, especially at T1.

One-way repeated ANOVAs revealed significant large time effects for emotional well-being [Wilks' λ = 0.65, *F*_(1, 18)_ = 9.64, *p* = 0.006, $\eta_{p}^{2}$ = 0.35], and expressive suppression [Wilks' λ = 0.74, *F*_(1, 18)_ = 6.26, *p* = 0.022, $\eta_{p}^{2}$ = 0.26]. Specifically, emotional well-being levels increased, and the use of expressive suppression decreased from the start of the program to its end.

### Brief discussion

The aims of Study 2 were to investigate the psychological effects of an adapted physical activity (APA) group training program, specifically tailored to the needs of older persons with osteoporosis. Attendance rate to the 14-week APA program was very high, despite the intensive training including two sessions per week. On average, participants reported overall good levels of well-being at T0; none was languishing based on the categorical diagnosis of mental health; they reported a more frequent use of reappraisal compared to suppression strategies of emotion regulation. At T1 participants reported significant improvements in emotional well-being, as well as a significant reduction in the use of suppression strategies of emotion regulation. In addition, the percentage of flourishing participants almost doubled.

These findings are consistent with a recent study investigating physical activity programs addressed to persons with osteoporosis, and showing improvements in both emotional and eudaimonic well-being dimensions (Mack et al., 2017). The specific contribution of this study consists in the evidence of a positive change in emotion regulation, with a significant decrease in participants' use of suppression. This result is consistent with previous research highlighting the role of socialization in promoting emotional disclosure and sharing, thus fostering an emotion-focused, approach type coping strategy (Stanton et al., 2000). This strategy is particularly adaptive in conditions of physical frailty, and overall in situations characterized by uncertainty and low control (Terry and Hines, 1998), such as the risk of falls in osteoporosis (Giangregorio et al., 2015).

## General discussion

This research was aimed at evaluating the effects of two training programs of adapted physical activity (APA) addressed to older adults—PoliFit and OsteoFit—on participants' hedonic and eudaimonic well-being, and emotion regulation strategies. The overall aim of the programs was to promote healthy aging and prevent physical and functional decay. While PoliFit was designed for globally healthy individuals, OsteoFit specifically addressed the needs of people with osteoporosis, targeting fall prevention through improvement of balance. Activities of both programs were performed in groups, consistent with the view of APA as an opportunity for socialization and bio-psycho-social well-being promotion (Mack et al., 2017).

The vast majority of participants in both studies were women, and around half of them reported living alone. Although most participants described engagement in structured free time activities, their life habits were substantially sedentary. The practice of sports was reported by a small minority in both studies, and it was limited in frequency.

As concerns the assessment of well-being before the training, the majority of participants in both studies reported moderate levels of mental health along the flourishing-languishing continuum; the percentages of flourishing people at T0 were comparable with those reported in a recent study conducted among Italian adults (Petrillo et al., 2015), while only one languishing participant was detected in Study 2, compared with 10% identified by Petrillo and colleagues. As concerns the different components of well-being, values were on average lower than those detected in some studies (Lamers et al., 2011; Petrillo et al., 2015) but higher than those reported in other ones (Keyes, 2005; Keyes et al., 2008); anyway, comparisons are problematic because no extensive data are available for MHC-SF in the aging population. In both studies, the lowest scores were detected for social well-being, in line with results from samples of Italian and South African adults (Keyes et al., 2008; Petrillo et al., 2015), and with international findings highlighting that community and society are the life domains in which adults across countries, especially in individualistic nations, report the lowest level of happiness and meaningfulness (Delle Fave et al., 2016).

In both studies, values of satisfaction with life before the training were aligned with findings obtained from older US adults (Pavot and Diener, 2009). As concerns emotion regulation, participants in both studies reported a more frequent use of the adaptive strategy of cognitive reappraisal compared to suppression.

The comparison between results obtained before and after the training programs highlighted the positive outcomes of both PoliFit and OsteoFit on well-being, especially at the emotional level. Participants in both studies reported an improvement in emotion regulation, with a significant increase in the use of cognitive reappraisal among PoliFit participants, and a significant decrease in suppression, matched with an increase in emotional well-being, among people attending OsteoFit.

These results are relevant, as emotion regulation is a crucial resource in aging. It allows individuals to successfully deal with the challenges and limitations imposed by the physiological aging process, and it is consistent with the Selection-Optimization-Compensation model (Baltes, 1997): By selecting affordable demands to cope with, optimizing the available skills and resources through the engagement in the pursuit of realistic goals, and compensating limitations with external supports or alternative behaviors, individuals may attain successful aging and preserve a positive view of themselves and the surrounding context. At the emotional level, older adults achieve well-being by selecting and optimizing particular emotion regulation processes to compensate for changes in internal and external resources (Urry and Gross, 2010). In particular, positive reappraisal strategies allow aging persons to adaptively face constraints and limitations, through the acknowledgment of related negative emotions and the engagement in psychological and behavioral pathways leading to the identification of adequate and realistic problem solving strategies.

An important resource in this process is represented by social interactions. The two APA programs were organized in group sessions, allowing people to interact with each other for a relatively long period on a regular weekly basis, and to disclose their experience of the challenges and opportunities related to the training activities. As highlighted by several studies, supportive social relations, especially among peers and people sharing similar experiences, promote the expression of emotions and their effective elaboration (Pennebaker, 1997) through an approach type coping process (Terry and Hines, 1998; Stanton et al., 2000). It is therefore possible that the experience of group training with other people sharing the same life stage and daily life challenges allowed PoliFit participants to increase their ability to positively appraise challenges and emotionally laden situations, and OsteoFit participants to overcome the tendency to suppress negative emotions.

Overall, the beneficial effects of the programs are in line with studies showing the positive role of active leisure and physical activity in promoting subjective and emotional well-being (Li et al., 2009; Kuykendall et al., 2015; Ku et al., 2016). As highlighted in a longitudinal study conducted in Finland (Lampinen et al., 2006), mental well-being in later life is associated with higher physical activity, better health and better mobility status; all these aspects should become targets for preventive measures. The adequacy of the training programs presented in this work is confirmed by a systematic review analyzing exercise based interventions designed for sedentary as well as frail older people, showing the effectiveness of a group-based approach, with weekly attendance (Windle et al., 2010). The review findings suggested the potential of this kind of interventions in fostering mental well-being, and their favorable cost-effectiveness ratio.

### Research strengths and limitations

The major strength of the present study is the longitudinal investigation of multiple dimensions of mental well-being, as well as emotion regulation strategies, among older adults before and after their participation in two newly designed training programs aimed at promoting healthy aging (Study 1) and at improving health and preventing falls in a frailty condition like osteoporosis (Study 2). The combination of different measures allowed for exploring different domains of mental health—emotional, psychological, and social—within a conceptual framework considering well-being not as the absence of disorders or pathological symptoms (as it is often operationalized in the biomedical research context), but as the presence of positive indicators. The assessment of well-being before and after the programs allowed for detecting their positive psychological outcomes, in terms of adaptive emotion regulation and emotional well-being. To the best of our knowledge, these studies are among the few ones assessing this kind of training programs and through this set of instruments in the Italian aging population.

This research has several limitations as well. In both studies, the lack of a control group—comprising, for example, older adults involved in non-physical group activities or in individual physical practice—does not allow to clearly distinguish the contribution of socialization to participants' improvement in emotion regulation from the contribution of the specific activity program. Moreover the samples were small, and they were almost completely comprised of women. Participants were independent in ADL, thus not reflecting the range of possible autonomy conditions characterizing the aging process; the inclusion of participants with lower levels of autonomy, besides requiring different typologies of adapted physical activities, could provide different psychological outcomes. Participants in both studies were Italian citizens of a metropolitan city, thus belonging to a specific socio-cultural context: While this feature increases reliability in the comparison of results between the two samples, it prevents from generalizing results to other countries, characterized by different healthcare and welfare systems, different family organization, and different views of aging.

### Future directions

Findings from the two studies presented here suggest that mental well-being in later life could be supported and improved through the group practice of physical activity. Due to the limitations reported above, these results cannot be considered as conclusive. Nevertheless, they can pave the way to further studies aimed at investigating the role of different structured leisure activities, performed individually or collectively, in the emotional well-being of older persons with and without physical frailties. Comparing the findings across activities and social settings may shed light on the specific contribution provided to well-being by socialization and by activity type. Researchers and practitioners are increasingly acknowledging the importance of promoting well-being in conditions of physical frailty and along the changes accompanying the aging process. The loss of primary control in several life domains characterizing this life stage may be successfully compensated by the development of psychological resources such as wisdom, and by the strengthening of adaptive regulatory strategies. These assets are shaped by individual and contextual factors; to this purpose, health professionals working with older people should support their potential for positive reappraisal of their own conditions, at the same time promoting competence building based on their perception of mastery and self-acceptance. These resources represent an important psychological capital that can be usefully exploited in the context of programs addressing physical health. Research in the health domain should more systematically explore these dimensions, overcoming the still predominating deficit-focused approach.

## Author contributions

AD and MB provided substantial contributions to the conception and design of the work; the acquisition, analysis, and interpretation of data; drafting the work and revising it critically for important intellectual content; final approval of the version to be published; and agreement to be accountable for all aspects of the work in ensuring that questions related to the accuracy or integrity of any part of the work are appropriately investigated and resolved. As concerns the other co-authors, GB and DM provided substantial contributions to the design of the training programs and to data collection, and they implemented the programs together with CR; EB actively participated in data coding and storing; all these co-authors also participated in data interpretation; critical revision of the work at the conceptual level; final approval of the version to be submitted; and agreement to be accountable for all aspects of the work in ensuring that questions related to the accuracy or integrity of any part of the work are appropriately investigated and resolved.

### Conflict of interest statement

The authors declare that the research was conducted in the absence of any commercial or financial relationships that could be construed as a potential conflict of interest.

## Footnotes

^1^C.A.M. (Centers for Multifunctional Aggregation) are facilities provided by the municipality of Milan in which city residents of any age group can attend a variety of activities, including social, leisure and sports events, as opportunities to be part of the community.

^2^Participants were also asked about their marital status. We did not report this information because only 20 out of 39 of them answered this question.

^3^Participants were also asked about their marital status. We did not report this information because only 6 out of 19 participants answered this question.
